# Acute LPS exposure enhances susceptibility to peripheral prion infection

**DOI:** 10.1038/s41598-025-94003-3

**Published:** 2025-03-21

**Authors:** Reiss Pal, Charlotte M. Thomas, Khalid Salamat, Stephen J. Jenkins, Barry M. Bradford, Neil A. Mabbott

**Affiliations:** 1 The Roslin Institute & Royal (Dick) School of Veterinary Studies, University of Edinburgh, Easter Bush, Midlothian, UK; 2 Queens Medical Research Institute, University of Edinburgh Centre for Inflammation Research, Edinburgh, UK

**Keywords:** Prion diseases, Transmissible spongiform encephalopathies, Disease susceptibility, Macrophage, LPS, Spleen, Peritoneal cavity, Infectious diseases, Prion diseases

## Abstract

After peripheral infections, the initial accumulation of prions within secondary lymphoid tissues is essential for the transmission of disease to the brain. Macrophages are considered to sequester or destroy prions, but little was known of their impact on disease susceptibility after a peripheral infection. Inflammation in the peritoneal cavity can trigger the macrophage disappearance reaction, whereby the macrophages are temporarily contained within cellular aggregates on the mesothelium. We studied the impact of the bacterial lipopolysaccharide (LPS)-mediated macrophage disappearance reaction on susceptibility to an intraperitoneal prion infection. Intraperitoneal LPS injection significantly enhanced prion disease susceptibility approximately 100X when given 24–3 h before infection. The effects on disease susceptibility coincided with the reduced abundance of macrophages within the peritoneal cavity at the time of infection and the enhanced early accumulation of prions in the spleen. This suggests that the reduced recoverable abundance of macrophages in the peritoneal cavity following acute LPS-treatment, increased disease susceptibility by enhancing the initial propagation of the prions from site of exposure (peritoneal cavity) to the spleen from where they subsequently spread to the brain. Further studies may help identify novel macrophage-targeted treatments that can reduce susceptibility to peripherally acquired prion infections.

## Introduction

Prion diseases, or transmissible spongiform encephalopathies, are a unique group of sub-acute, infectious, neurodegenerative disorders which can affect humans and other mammalian species such as sheep, goats, cattle and cervids. Infectious prions comprise predominantly, if not entirely, of PrP^Sc^: mis-folded isomers of the host cellular prion protein^1,2^. The accumulation of prions within the central nervous system (CNS) during infection ultimately leads to the development of extensive neurodegeneration in affected regions of the brain. No effective treatments are currently available to treat prion diseases and infection is invariably fatal.

Many natural prion infections are acquired. For example, natural sheep scrapie, chronic wasting disease (CWD) in cervids, bovine spongiform encephalopathy (BSE) in cattle and variant Creutzfeldt-Jakob disease in humans (vCJD) are each considered to be orally acquired via consumption of food, pasture or other materials contaminated with prions (for review see:^3^). After peripheral infection the prions initially accumulate in the secondary lymphoid tissues such as the Peyer’s patches after oral exposure, and the spleen after intraperitoneal (IP) injection^4–8^. The early accumulation and replication of the prions within lymphoid tissues is essential for their efficient transmission to the brain, a process termed neuroinvasion^9–15^. Factors such as inflammation, co-infection with other pathogens or aging can each affect susceptibility to a peripherally-acquired prion infection. This may be mediated by modulating the efficiency of the propagation of the prions from the site of exposure into the draining lymphoid tissues (for review see:^16^). For example, M cells play an important role in the initial transportation of prions across the gut epithelium into the Peyer’s patches after oral exposure^17,18^. In aged mice the density of M cells in the gut epithelium is reduced^19^and coincides with the reduced susceptibility of aged mice to oral prion infection^20^.

Acute pro-inflammatory stimulation or bacterial infection in the peritoneal cavity can trigger the macrophage disappearance reaction, whereby the macrophages within it are transiently no longer retrievable as they are contained within temporary cellular aggregates that attach to the mesothelium^21–23^. Although macrophages are considered to sequester or destroy prions^24–28^, little was known of the effects of reduced macrophage abundance on susceptibility to a peripheral prion infection. To address this issue experiments were designed to study the impact of acute bacterial lipopolysaccharide (LPS)-mediated induction of the macrophage disappearance reaction on susceptibility to prion infection via the peritoneal cavity. A greater understanding of how the effects of acute inflammation on macrophage abundance at the site of infection may increase susceptibility to peripherally-acquired prion infections, may help identify important risk factors or novel treatments that can reduce susceptibility to peripherally acquired prion infections.

## Materials and methods

### Mice and prion infection

Male and female C3H/HeN mice (6–10 weeks old; Charles River, Margate, UK) were used in this study and maintained under specific pathogen-free conditions. These mice were used in many studies alongside a wide range of other wild-type mouse strains at our former institution (Neuropathogenesis Unit, Edinburgh, UK) to compare the impact of host genotype (mouse background) on prion disease pathogenesis^29,30^. These mice share the same *Prnp* genotype (encoding PrP^C^) as other common wild-type mouse strains such as C57BL/6 (*Prnp*^a^, previously known as *Sinc*^S7^)^31^.

Mice were randomly assigned to cages and groupings before the onset of each study. Mice were given a single intraperitoneal (IP) injection of 20 µl of brain homogenate prepared from a mouse terminally infected with mouse-passaged ME7 scrapie prions. The relative doses of prions used in each experiment are described in the text and ranged from a 0.0001–1.0% (weight/vol) dilution of scrapie brain homogenate. Following injection with prions the mice were coded and assessed blindly at daily intervals for the clinical signs of prion disease, and culled at a standardised humane end-point upon the development of terminal clinical signs of prion disease^32^. Clinically negative survivors were culled at the end of the experiment.

Brains were collected from all mice and the presence or absence of clinical prion disease was confirmed by histopathological assessment of the abundance and distribution of the spongiform vacuolar degeneration in paraffin-embedded, haematoxylin & eosin (H&E) stained sections^33^. The severity of the prion disease‐specific vacuolation was scored in nine grey matter and three white matter areas using a 0–5 scale: G1, dorsal medulla; G2, cerebellar cortex; G3, superior colliculus; G4, hypothalamus; G5, thalamus; G6, hippocampus; G7, septum; G8, retrosplenial and adjacent motor cortex; G9, cingulate and adjacent motor cortex; W1, inferior and middle cerebellar peduncles; W2, decussation of superior cerebellar peduncles; and W3, cerebellar peduncles.

### Ethics statement

The in vivo mouse experiments were reviewed and approved by the Neuropathogenesis Unit’s (Edinburgh) or University of Edinburgh’s ethics committees. All the methods and procedures used in the in vivo experiments within this study were performed in accordance with the relevant guidelines and regulations of a UK Home Office Project License. Each set of experiments was designed and reported as described by the ARRIVE guidelines^34^. Care was provided throughout the experiments in order to minimize harm and suffering. At the end of the experiments the animals were humanely culled by cervical dislocation.

### Bacterial lipopolysaccharide (LPS) treatment

At the times indicated the mice were given a single IP injection with LPS derived from Salmonella Typhosa (L6386, Sigma, Poole, UK) in sterile PBS. The doses of LPS used in each experiment are indicated in the text. Untreated mice or mice treated with sterile PBS alone were used as controls.

### Histopathology & immunohistochemistry

Formalin-fixed paraffin-embedded brain sections and periodate-lysine-paraformaldehyde fixed paraffin-embedded spleen sections (thickness 6 μm) were deparaffinised and pre‐treated by autoclaving in target retrieval solution (Dako) at 121 °C for 15 min. Endogenous peroxidases were quenched by immersion in 4% hydrogen peroxide in methanol for 10 min. Microglia were detected by immunostaining with rabbit anti-allograft inflammatory factor-1 (AIF1; 1/1000 dilution) polyclonal antibody (Wako, Japan). Astrocytes were detected using rabbit anti-glial fibrillary acidic protein (GFAP; 1/2000 dilution) polyclonal antibody (Agilent Dako). For the detection of PrP in the brain, prior to immunostaining the sections were immersed in 98% formic acid for 10 min, and the PrP subsequently detected by immunostaining with mouse anti-PrP monoclonal antibody (mAb) BH-1^35^(1/6000 dilution). The presence of PrP in spleen sections was detected by immunostaining with rabbit 1B3 PrP-specific polyclonal antiserum (1/4000 dilution)^36^. Follicular dendritic cells were detected in the spleen using anti-CD21/35 mAb (clone 7G6; BD Biosciences; 1/100 dilution). Macrophages were detected in the spleen using F4/80 mAb (clone CI: A3-1; Bio-Rad Laboratories; 1/100 dilution). Following addition of biotinylated secondary antibodies, immunostaining was revealed using the Vectastain avidin-biotin complex (ABC) kit (Vector Laboratories) with 3,3′-diaminobenzidine tetrahydrochloride (DAB; Sigma) as a substrate on brain sections or Vector NovaRED^®^substrate (Vector Laboratories) on spleen sections. Haematoxylin was used as a counterstain to detect cell nuclei, and imaged on a Ni.1 Brightfield Compound upright microscope (Nikon) using 4x/10x/20x/ air lenses, a 105c colour camera (Zeiss) and Zen 2 software (Zeiss) for image capture. Paraffin-embedded tissue (PET) immunoblot^37^ was used to confirm the presence of prion disease-specific, relatively proteinase K-resistant, PrP^Sc^ and the membranes immunostained with 1B3 polyclonal anti-serum to detect PrP. Following addition of alkaline phosphatase-conjugated secondary antibody the immunostaining was revealed with Sigma Fast 5-bromo-4-chloro-3-indolyl phosphate/nitro blue tetrazolium (NBT-BCIP; Sigma), and imaged using a Lumar.V12 stereoscope (Zeiss) with a 0.8x objective lens, and Zen 2 software for image capture.

### Real-time quaking-induced conversion (RT-QuIC)

Full-length bank vole recombinant PrP^C^(recPrP; residues 23–230; GenBank accession number: AF367624) was used as the substrate for the RT-QuIC reactions (Orru et al., 2015). The recPrP expression construct was expressed in E. coli Rosetta™ 2(DE3) cells (Merck) and purified as previously described^38^. Spleen homogenates were diluted to 1% (vol/vol) in PBS (pH 7.4) supplemented with 0.025% SDS, and subsequently used in RT-QuIC reactions as previously described (Orru et al., 2015) with modifications. Briefly, aliquots (95 µl) of RT-QuIC reaction buffer (PBS supplemented with 170 mM NaCl, 1 mM EDTA and 10 µM Thioflavin T (ThT)) containing 0.1 mg/ml recPrP substrate, were added to the wells of a black, clear-bottomed 96-well plate (Nunc). Individual wells were seeded with 5 µl of the appropriate spleen homogenate from a blinded panel of samples, alongside known positive and negative controls (*n* = 4 replicates/sample). Plates were incubated at 42ºC in a POLARstar Omega plate reader (BMG Labtech) with 1 min cycles of shaking (700 rpm, double orbital) followed by 1 min rest. ThT fluorescence was recorded every 15 min (450 nm excitation, 480 nm emission, gain 2000) for a total of 90 h (equivalent to 360 cycles). Samples were considered positive for seeding activity if the mean ThT fluorescence (from *n* = 4 replicate reactions) exceeded a pre-determined threshold within 90 h (threshold = twice the mean fluorescence from all negative control measurements, equivalent to 123,466 RFU).

### Flow cytometry

Peritoneal cells collected by lavage were resuspended in buffer (Dulbecco’s PBS, 1mM HEPES and 2mM EDTA), treated with Live-Dead Zombie Aqua –BV510 stain (Biolegend) for 10 min and non-specific antibody binding was subsequently blocked using rat anti mouse CD16/32 antibody mAb (BD Biosciences, 1:300 dilution) for another 10 min. Cells were washed in FACS buffer (containing 2mM EDTA/0.5% BSA in PBS) at 300 x *g* for 5 min and then incubated with the directly fluorescently-labelled primary antibodies listed in Table 1 for 30 min at 4 °C. Cells were then fixed using the eBioscience Foxp3 staining buffer (Invitrogen). Data were acquired using a FACS LSRFortessa (BD) and analysed using the FlowJo software (version 10.5.0, Treestar). Doublet events (identified through forward scatter area vs. forward scatter height plot) and dead cells (identified by Live/Dead Zombie Aqua) were excluded in the analysis^39^. The gating strategy that was used is shown in Fig. 6A and is equivalent to that used by Vega-Perez and colleagues in their study of the impact of *E. coli *infection on resident macrophages in the peritoneal cavity^23^.

**Table 1:** Monoclonal antibodies (mAb) used for flow cytometry.

Target molecule	mAb clone	Source	Fluorochrome	Dilution
CD3	OKT3	Biolegend	PE	1:200
CD19	HIB19	Biolegend	PE	1:200
CD11b	M1/70	Biolegend	PE-Dazzle (CF594)	1:400
CD11c	AFS98	Biolegend	APC-CY7	1:200
CD102	3C4	Biolegend	FITC	1:200
CD115	ASF98	Biolegend	APC	1:200
Ly6C	HK1.4	Biolegend	BV711	1:400
Ly6G	1A8	Biolegend	PE	1:200
MHCII (IA-IE)	M5/114.15.2	Biolegend	AF700	1:400
Siglec-F	E50-2440	BD Biosciences	PE	1:100

### Statistical analyses

Statistical significance between groups was tested using Prism 7.0 software (GraphPad, San Diego, United States). Data were first checked for normal distribution using the Shapiro-Wilk test. Differences between groups were compared using One-Way ANOVA and post-hoc Tukey multiple comparisons tests. Where indicated, survival curve data for prion infected mice in each treatment group were compared by Log-rank (Mantel-Cox) test, and Reed and Meunch analysis. Flow cytometry data were log10 transformed before analysis. Data are presented as mean ± SD, unless indicated otherwise. Data points represent individual animals. Values of *P* < 0.05 were accepted as significant. *, *P* < 0.05; **, *P* < 0.01; ***, *P* < 0.001; ns, not significantly different.

## Results

### LPS treatment enhances prion disease susceptibility when given 3 h before infection

Groups of male and female mice were first given a single IP injection of LPS (10 µg) and subsequently injected IP with ME7 scrapie prions. The relative timings between the LPS and prion infections are shown in Table 2. A separate group of mice was injected with prions and subsequently injected with LPS 3 h later. Untreated mice, or mice treated with PBS 3 h before IP prion infection were used as controls.

**Table 2:** Influence of intraperitoneal (IP) LPS injection on susceptibility to IP prion infection.

Treatment^a^	Timing of treatment relative to prion infection	Clinical disease incidence^b^	Mean survival time (Days ± SD)^c^	Significance v. PBS-treated^d^
LPS	−6 days	4/8	330 ± 18, *309*,* 332*,* 337*,* 351*, 4X > 644	ns
LPS	−48 h	7/10	326 ± 13, 3X > 644	ns
LPS	−24 h	9/10	323 ± 9, 1X > 644	0.0156
LPS	−3 h	10/10	315 ± 10	0.001
LPS	+ 3 h	2/10	*325*, *360*, 8X > 644	ns
PBS	−3 h	4/9	330 ± 18, *309*,* 337*,* 337*,* 344*, 5X ≥546	n/a
Untreated	n/a	2/9	*318*,* 322*, 7X ≥ 644	ns

^a^PBS or LPS (10 µg/mouse) administered by IP injection.

^b^Clinical disease incidence = number with clinical and histopathologically confirmed prion disease/number injected IP with prions.

^c^Individual survival times are shown in italics. NX > 644, indicates the number of mice that were free of the signs of prion disease up to at least this time after IP injection.

^d^Survival analysis by Log-Rank test. ns, not significant.

**Fig. 1:**
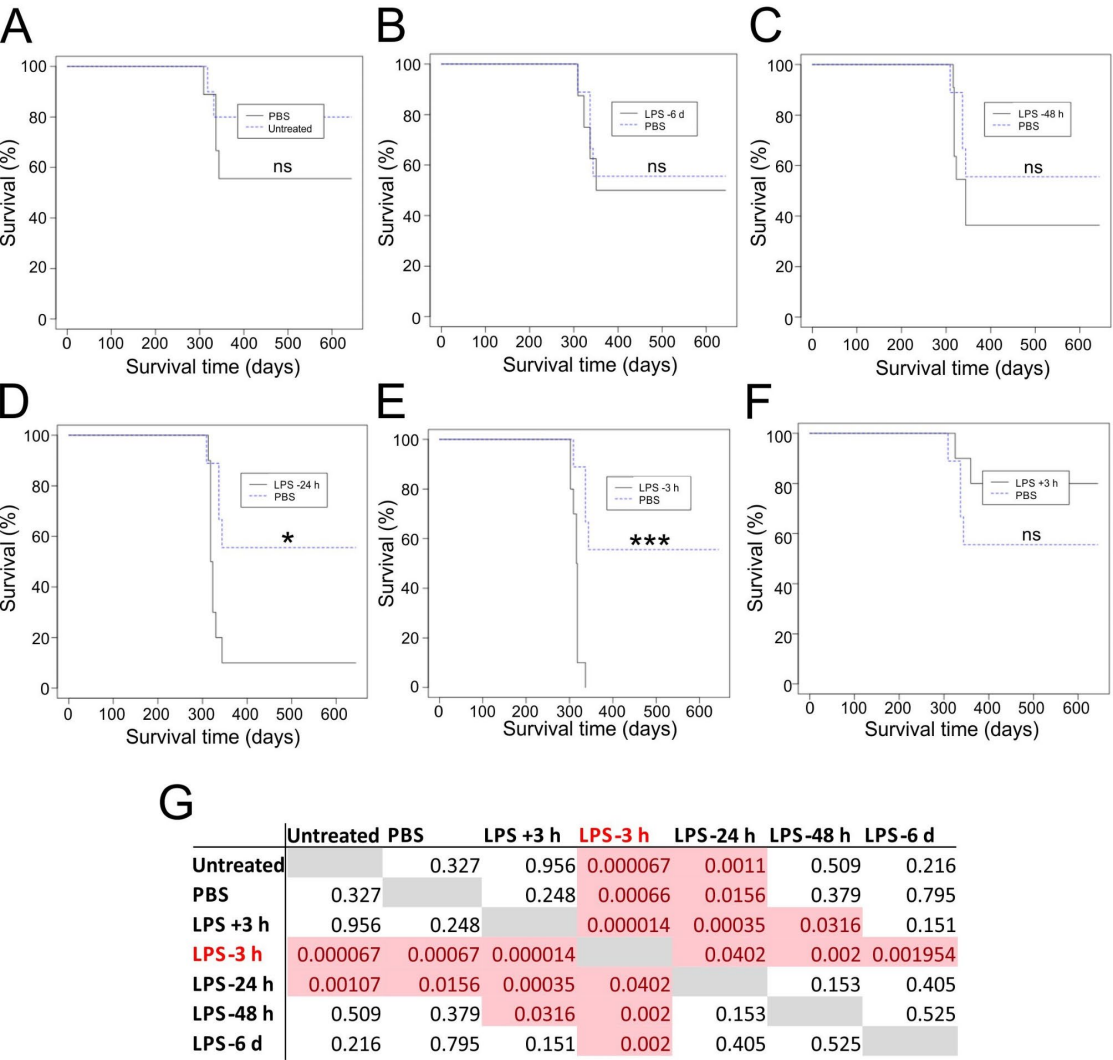
LPS treatment enhances prion disease susceptibility when given 3 h before intraperitoneal (IP) infection. Survival plots showing the impact of LPS treatment on prion disease susceptibility. Groups of mice (*n* = 8–10) were given a single IP injection with a limiting dose of prions (0.01%) and also injected IP with LPS (10 µg) or PBS (control) at the following intervals in relation to the timing of the prion infection: **(A)** untreated (prions alone); **(B)** −6 days; **(C)** −48 h; **(D)** −24 h; **(E)** −3 h; **(F)** + 3 h. *, *P* < 0.05; ***, *P* < 0.001; ns, not significant; Log-rank (Mantel-Cox) test. **(G)** Survival rate statistics for all treatment group comparisons.

The mice were injected with a limiting dose of ME7 scrapie prions (0.01% [wt/vol] dilution of brain homogenate prepared from a mouse with terminal prion disease). This dose of prions was used to enable any effects on disease susceptibility to be determined, as it causes clinical disease in < 100% of untreated C3H/HeN mice (Table 2). As anticipated, only a fraction of the untreated or PBS-treated control mice succumbed to clinical prion disease and the survival rates between each of these control groups were not significantly different (Fig. 1A; Table 2, Log-Rank test). Treatment with LPS 6 days or 48 h before IP prion infection also did not affect disease susceptibility compared to PBS-treated controls (Fig. 1B&C; Table 2). In contrast, prion disease susceptibility was significantly increased when the mice were treated with LPS either 24–3 h before IP prion infection (Fig. 1D&E; Table 2). All the mice injected with LPS 3 h before prion infection succumbed to clinical prion disease with a mean survival time of 315 ± 10 days (median, 317; *P* < 0.001, Log-Rank test; Table 2). The impact of LPS on disease susceptibility was limited to the hours immediately before IP prion injection. When LPS treatment was given as soon as 3 h after prion injection disease susceptibility was not affected compared to PBS-treated and untreated controls (Fig. 1F; Table 2). The survival rate statistics for all treatment group comparisons are provided in Fig. 1G. Together, these data show that a single IP injection with LPS significantly enhanced prion disease susceptibility when given between 24 and 3 h before infection. However, the effects of LPS on prion disease were acute, since treatment as soon as 3 h after infection had no effect of disease susceptibility compared to controls.

**Fig. 2:**
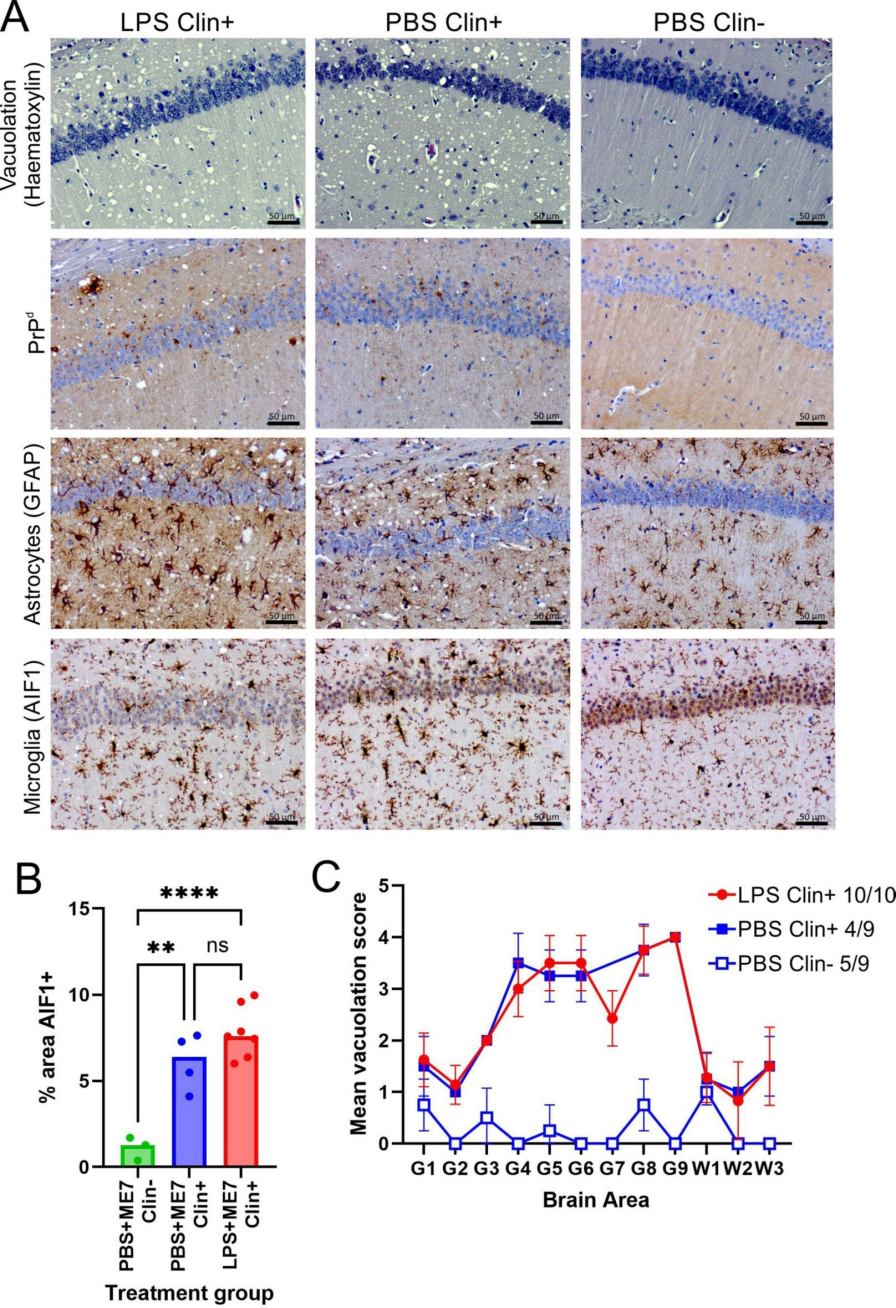
LPS-treatment 3 h before intraperitoneal (IP) prion infection does not affect the development of the neuropathological signs of CNS prion disease. Mice were first injected IP with LPS (10 µg, *n* = 10) or PBS (control, *n* = 9), and 3 h later injected IP with a limiting dose of prions (0.01%). Brains were collected at the terminal clinical stage or the end of the experiment at ≥546 days after injection. **(A)**, Representative images showing high levels of spongiform pathology (upper row), prion disease-specific PrP^d^ accumulation (brown, 2nd row), reactive astrocytosis (GFAP + cells, brown, 3rd row) and microgliosis (AIF1 + cells, brown, bottom row) in the hippocampus of the brains of clinically affected mice (Clin+) at the terminal clinical stage. No histopathological signs of prion disease were detected in the brains of the clinically negative (Clin-) survivors. Sections counterstained with haematoxylin to detect cell nuclei (blue). Scale bar, 50 μm. **(B)** The relative abundance of AIF1 + immunostaining in the brains of the clinically affected mice (Clin+) at the terminal clinical stage, or clinically negative (Clin-) survivors. **, *P* < 0.01; ***, *P* < 0.001; ns, not significant; Student’s t-test. **(C)** The severity and distribution of the spongiform pathology (vacuolation) within each brain from each treatment group was scored in the following gray matter and white matter regions: G1, dorsal medulla; G2, cerebellar cortex; G3, superior colliculus; G4, hypothalamus; G5, thalamus; G6,hippocampus; G7, septum; G8, retrosplenial and adjacent motor cortex; G9, cingulate and adjacent motor cortex; W1, inferior and middle cerebellar peduncles; W2, decussation of superior cerebellar peduncles; and W3, cerebellar peduncles. Data points represent mean ± SD.

Histopathology confirmed that the brains of all the mice that developed clinical disease in each group had the characteristic neuropathological signs of CNS prion disease. These included vacuolar pathology in the neuropil, disease-specific prion protein accumulation (PrP^d^), astrocytosis and microgliosis (Fig. 2A). No histopathological signs of prion disease were detected in any brains of the clinically negative mice (Fig. 2A). No significant difference in the abundance of AIF1 + immunostaining was detected in the brains of the clinically-affected control and LPS-treated mice (Fig. 2B). This suggested that IP LPS-treatment around the time of IP prion infection did not influence the density of AIF1 + microglia in the brain at the terminal, clinical stage of disease, consistent with our previous study^32^. The magnitude and distribution of the vacuolar pathology in the brains of the clinically-affected control and LPS-treated mice was also similar (Fig. 2C). This indicated that LPS-treatment around the time of IP prion injection did not affect the development of the neuropathology once the infection was established within the CNS. This conclusion is consistent with data in our previous study which showed that IP LPS treatment soon after prion infection directly into the brain by intracerebral injection had no impact on the subsequent development of CNS prion disease^32^. This indicated that the effects of LPS on disease susceptibility were due to effects on cells in the peritoneal cavity rather than in the CNS.

### LPS treatment enhances prion disease susceptibility approximately 100X

**Fig. 3:**
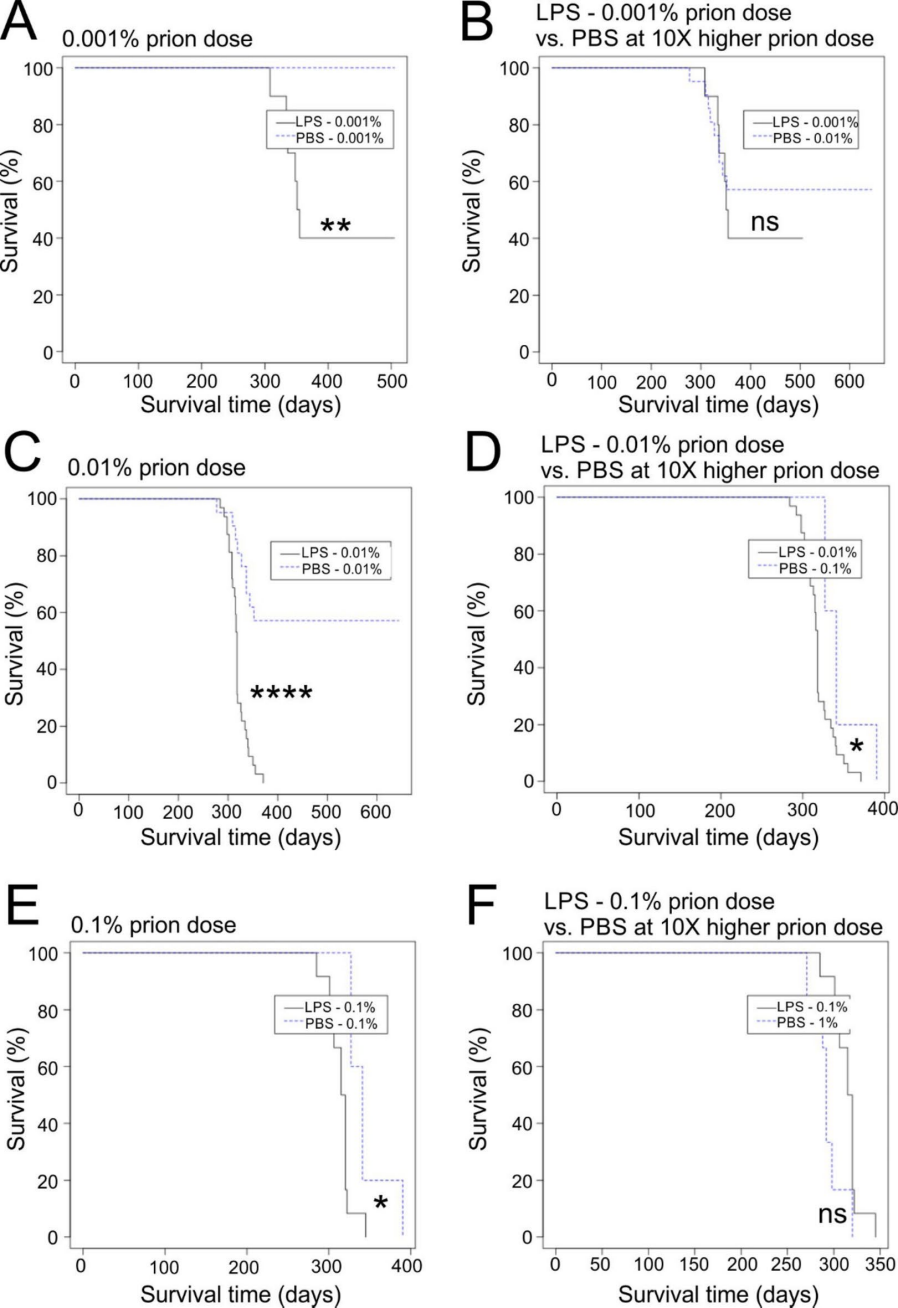
LPS treatment 3 h before intraperitoneal (IP) prion infection enhances disease susceptibility. Groups of mice (*n* = 5–32) were treated with LPS (10 µg) or PBS (control) 3 h before IP injection with a range of prion doses ranging from 1–0.001% and survival times recorded. **(A**,** C**,** E)** Survival plots for LPS and PBS-treated mice injected IP with a **(A)** 0.001% dose, **(C)** 0.01% dose, or **(E)** 0.1% dose of prions. **(B**,** D**,** E)** Survival plots showing LPS-treated mice injected with a **(B)** 0.001% dose, **(D)** 0.01% dose, or **(F)** 0.1% dose of prions, compared to PBS-treated controls injected with 10X higher doses of prions. *, *P* < 0.05; **, *P* < 0.01; ****, *P* < 0.0001; ns, not significant; Log-rank (Mantel-Cox) test.

To study the impact on disease susceptibility further, groups of mice were treated with LPS (10 µg) or saline (control) 3 h before IP injection with different prion doses ranging from 1 to 0.0001%. Across the dilution series, survival rates and clinical disease incidences in LPS-treated mice were typically equivalent to those observed in control-treated mice infected with higher doses of prions (Table 3). For example, no significant differences in survival rates were observed between LPS-treated mice injected with a 0.001% dose of prions, compared to PBS-treated controls injected with a 10X higher dose (0.01% dose of prions; Log-Rank test; Table 3; Fig. 3A&B). Similar effects were observed for LPS-treated mice injected with either a 0.01% or 0.1% dose of prions, compared to PBS-treated controls injected with higher doses (Table 3; Fig. 3C-F). Although all the PBS-treated mice succumbed to clinical prion disease when infected with a much higher dose of prions (0.1%) as expected, the survival times for the LPS-treated mice infected with either a 10X lower (0.01%) or equivalent dose of prions (0.1%) were significantly shorter (Log-Rank test; Table 3; Fig. 3D&E). Further comparisons of disease incidences across the dilution series by Reed and Meunch analysis revealed a 2 log increase in titre in the LPS-treated mice compared to PBS-treated controls: PBS-treated mice, log_10_ID_50_10-^3.75^; LPS-treated mice log_10_ID_50_ 10^−5.77^. This indicated that LPS-treatment increased prion disease susceptibility by approximately 100X.

**Table 3:** Intraperitoneal LPS exposure increases susceptibility to peripherally-acquired prion infection.

Treatment^a^	Prion dose (wt/vol)^b^	Clinical disease incidence^c^	Mean survival time (Days ± SD)^d^	Significance v. PBS-treated^e^
LPS	1%	11/11	306 ± 28	ns
PBS	1%	6/6	294 ± 16	
LPS	0.1%	12/12	314 ± 15	0.0126
PBS	0.1%	5/5	345 ± 26	
LPS^f^	0.01%	32/32	314 ± 18	7 x 10^-7^
PBS^f^	0.01%	9/21	324 ± 23, 12 × 412–644	
LPS	0.001%	6/10	329 ± 17, 4X > 505	0.00625
PBS	0.001%	0/9	9X > 504	
LPS	0.0001%	0/11	11X > 504	ns
PBS	0.0001%	0/8	8X > 504	

^a^PBS or LPS (10 µg) administered by intraperitoneal (IP) injection 3 h before IP injection with prions.

^b^Mice injected with 20 µl of X% dilution of brain homogenate prepared from a mouse with terminal prion disease.

^c^Clinical disease incidence = number with clinical and histopathologically confirmed prion disease/number injected IP with prions.

^d^NX > 504, indicates the number of mice that were free of the signs of prion disease up to at least this time after IP injection.

^e^Survival analysis by Log-Rank test.

^f^Combined data from three independent experiments. All other data are derived from an individual experiment.

### Low dose LPS-treatment enhances prion disease susceptibility

**Fig. 4:**
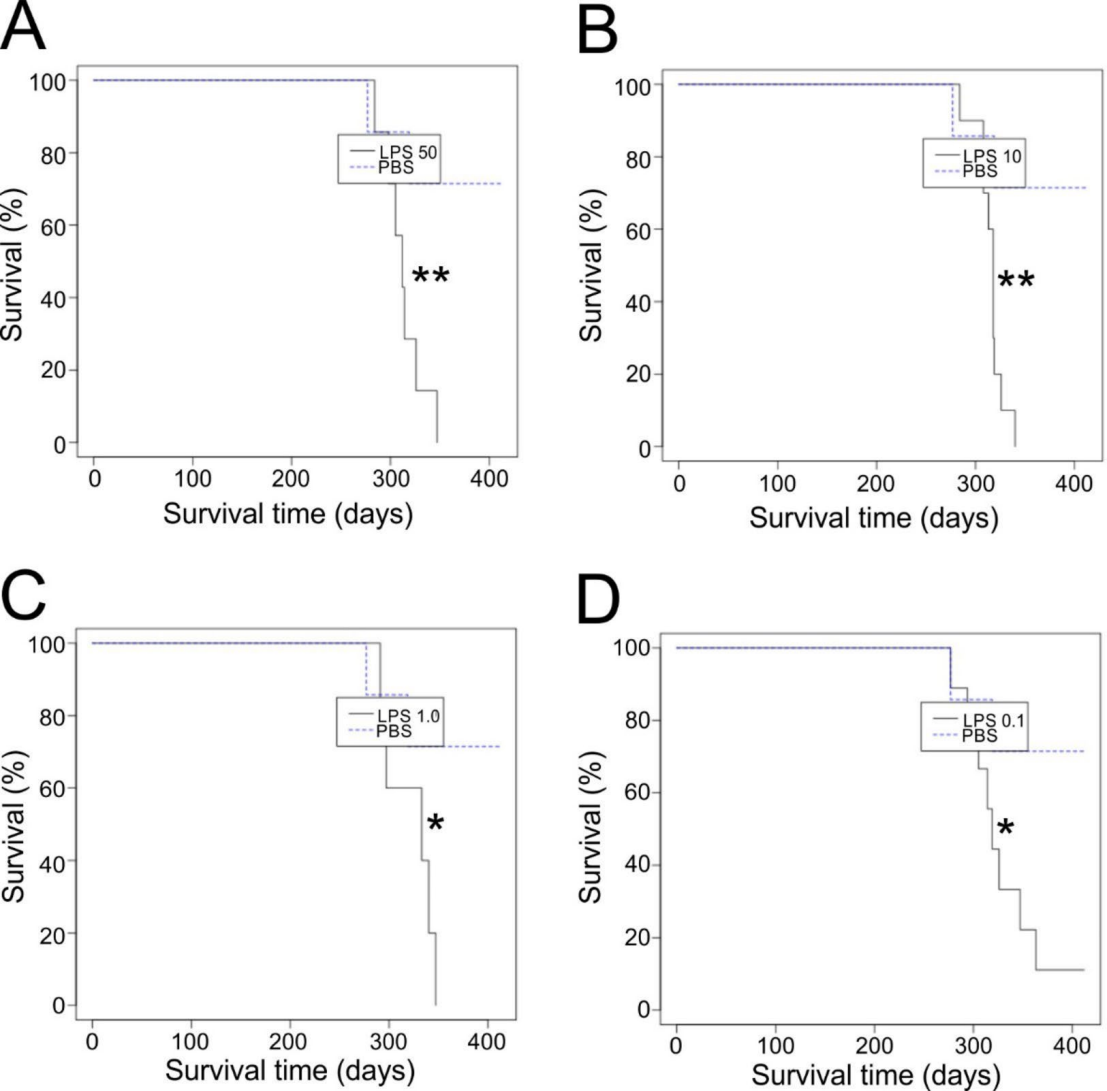
Low dose LPS-treatment enhances prion disease susceptibility. Groups of mice (*n* = 6–10) were first given a single intraperitoneal (IP) injection with a range of LPS doses from 50 µg to 0.1 µg, or PBS (control), and 3 h later injected IP with a limiting dose of prions (0.01%). Survival plots for mice injected with either **(A)** 50 µg, **(B)** 10 µg, **(C)** 1.0 µg or **(D)** 0.1 µg LPS. *, *P* < 0.05; **, *P* < 0.01; Log-rank (Mantel-Cox) test.

We next determined whether prion disease susceptibility was influenced by the magnitude of the LPS dose. Groups of mice were first given a single IP treatment with a range of LPS doses between 0.1 and 50 µg, and 3 h later injected IP with a limiting dose of ME7 scrapie prions (0.01% brain homogenate). Mice treated with PBS 3 h before IP prion infection were used as controls. This analysis showed that each LPS dose was sufficient to significantly increase disease susceptibility compared to PBS-treated controls **(**Table 4; Fig. 4A-D). Furthermore, the survival rates of mice treated with the lowest dose of LPS used (0.1 µg) were similar to those of mice treated with higher doses of LPS (1–50 µg). Thus, a single IP treatment with a low dose of LPS (0.1 µg) 3 h before IP prion infection was sufficient to significantly increase disease susceptibility.

**Table 4:** Influence of LPS dose on susceptibility to peripheral prion infection.

Treatment^a^	LPS dose (µg)	Clinical disease incidence^b^	Mean survival time (Days ± SD)^c^	Significance v. PBS-treated^d^	Significance v. 0.1 µg LPS^d^
LPS	50	7/7	312 ± 20	0.0113	ns
LPS	10	10/10	315 ± 14	0.005	ns
LPS	1	5/5	315 ± 26	0.0379	ns
LPS	0.1	8/9	318 ± 28	0.0334	n/a
PBS	n/a	2/6	*277*, *391*, 4 × 412	n/a	0.0334

^a^PBS or LPS administered by intraperitoneal (IP) injection 3 h before IP prion injection.

^b^Clinical disease incidence = number with clinical and histopathologically confirmed prion disease/number injected IP with prions.

^c^Individual survival times are shown in italics. NX > 412, indicates the number of mice that were free of the signs of prion disease up to at least this time after IP injection.

^d^Survival analysis by Log-Rank test.

### LPS-treatment enhances the early accumulation of prions in the spleen

**Fig. 5:**
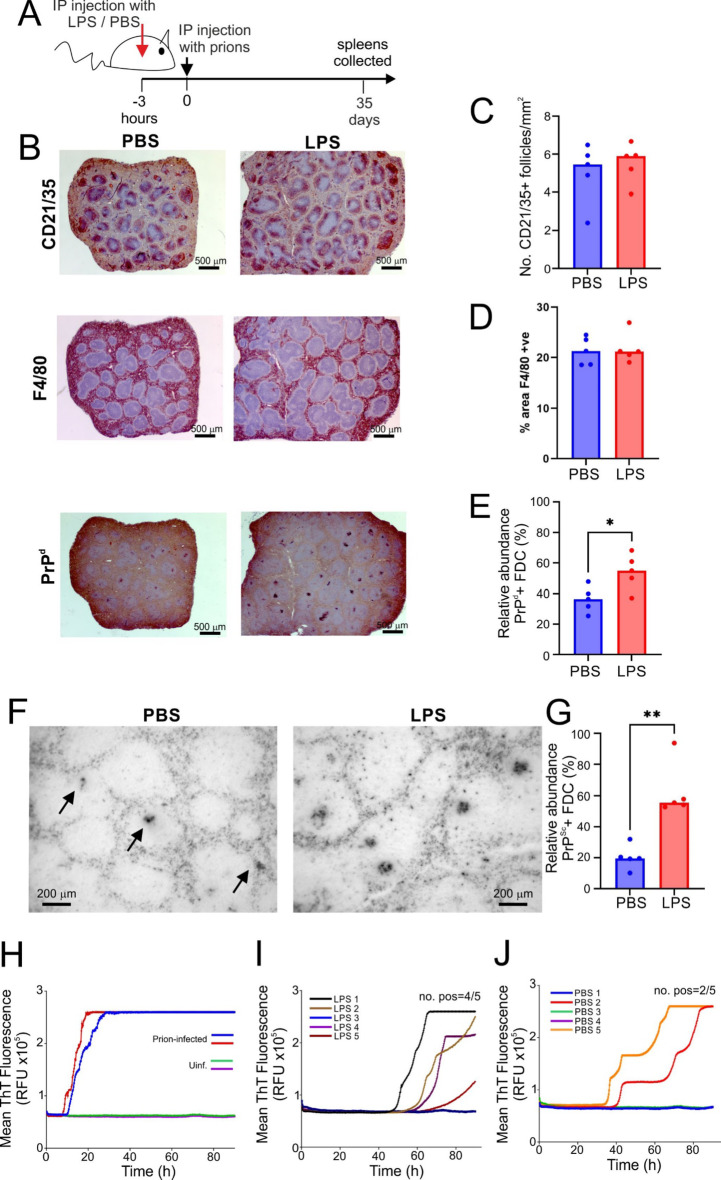
LPS-treatment enhances the early accumulation of prions in the spleen. **(A)** Experimental design. Groups of mice (*n* = 5) were first given a single intraperitoneal (IP) injection of LPS (10 µg) or PBS (control), 3 h later injected IP with a low dose of prions (1.0%) and spleens were harvested 35 days-post injection. **(B)** Immunohistochemical analysis of follicular dendritic cell (FDC)-associated (CD21/CD35^+^ cells, upper panels), F4/80 + macrophages (middle panels) and disease-specific prion protein (PrP^d^) accumulations (lower panels) in spleens of mice from each treatment group at 35 days post-injection with prions. Sections were counterstained with hematoxylin (blue). Scale bar = 500 μm. **(C)** LPS treatment did not affect the density of FDC networks in the spleen compared to PBS-treated controls. Data points represent individual mice. Bars, median. Not significantly different, Student’s t-test. (**D)** LPS treatment did not affect the abundance of F4/80 + immunostaining in the spleen compared to PBS-treated controls. Data points represent individual mice. Bars, median. Not significantly different, Student’s t-test. **(E)** The relative abundance of PrP^d^+ FDC networks (% positive networks) was increased in the spleens of LPS-treated mice compared to PBS-treated controls. *, *P* < 0.05, Student’s t-test. **(F)** PET blot analysis of adjacent sections confirmed the presence of relatively proteinase K-resistant prion disease specific PrP^Sc^ (black, arrows). Scale bar = 200 μm. **(G)** The relative abundance of PrP^Sc^+ FDC networks (% positive networks) was increased in the spleens of LPS-treated mice compared to PBS-treated controls. **, *P* < 0.01, Student’s t-test. **(H-J)** Relative prion seeding activities in spleens from each treatment group were quantified in vitro by RT-QuIC analysis. Individual traces represent spleens from individual mice. **(H)** Spleens from terminally infected mice and uninfected mice were used as positive and negative controls, respectively. **(I)** Spleens from LPS-treated mice and **(J)** PBS-treated mice collected 35 days after injection with prions. Data points derived from mean values from two sections/spleen.

After IP exposure the prions are initially propagated from the peritoneal cavity to the spleen where their accumulation and amplification upon stromal-derived follicular dendritic cells (FDC) is an essential requirement for their subsequent spread to the CNS^11,15,40^. To study the impact of LPS treatment on the early accumulation of prions in the spleen, groups of mice (*n* = 5) were first treated with LPS (10 µg) or PBS (control) and injected 3 h later with a low dose of prions (1.0%). Spleens were harvested 35 days later (Fig. 5A). Immunohistochemical analysis revealed that LPS-treatment did not affect the abundance of CD21/35 + FDC networks or F4/80 + macrophages in the spleen, as their density was similar to PBS-treated controls (Fig. 5B-D). However, the early relative abundance of FDC-associated prion disease-specific PrP accumulations (PrP^d^) was significantly increased in the spleens of LPS-treated mice compared to controls (Fig. 5B&E). Paraffin-embedded tissue (PET) immunoblot analysis confirmed that these FDC-associated aggregates contained relatively proteinase K-resistant prion disease specific PrP^Sc^ (Fig. 5F&G). Consistent with the histological data, the proportion of spleens containing detectable prion-seeding activity measured by RT-QuIC analysis (Wilham et al., 2010) was also increased in the LPS-treated mice at 35 days after infection compared to controls (Fig. 5H-J). Whereas 4 of 5 of the spleens from LPS-treated mice contained detectable prion-seeding activity (Fig. 5I), only 2 of 5 PBS-treated mice displayed this activity (Fig. 5J). Together, these data suggest that LPS treatment enhanced the early accumulation of prions in the spleen.

### LPS treatment reduces the abundance of peritoneal macrophages

Studies suggest that mononuclear phagocytes such as macrophages play a host protective role by engulfing and sequestering prions. For example, prion accumulation in the Peyer’s patches of orally-infected mice was increased following macrophage depletion^26^. Inflammation or bacterial infection in the peritoneal cavity can trigger the macrophage disappearance reaction, whereby the macrophages within it are transiently no longer retrievable as they are contained within cellular aggregates that attach to the mesothelium^21–23^. We therefore considered whether LPS-treatment increased the efficiency of the initial propagation of the prions from site of exposure (peritoneal cavity) to the spleen by similarly reducing the retrievable abundance of macrophages in the peritoneal cavity.

**Fig. 6:**
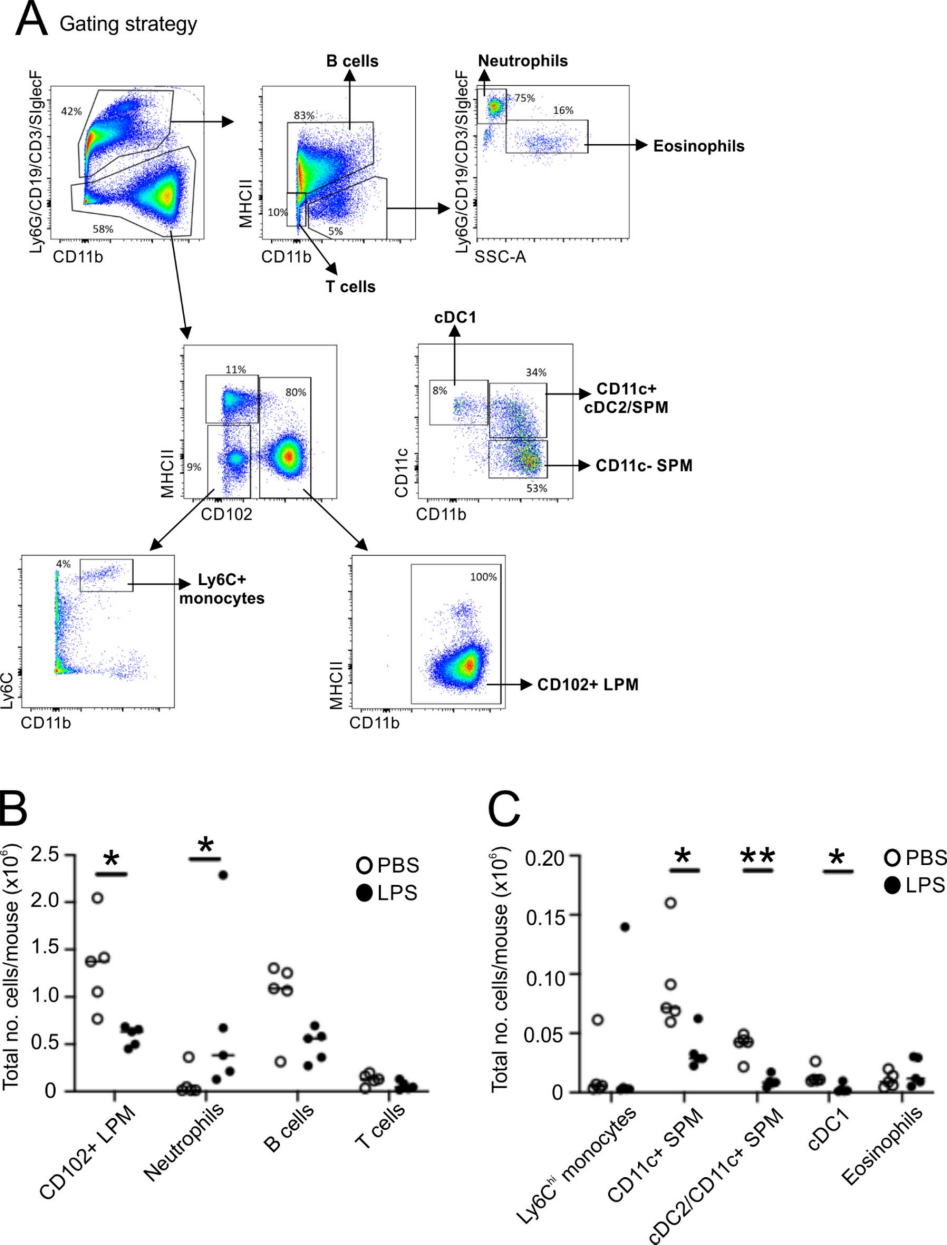
LPS treatment reduces the abundance of peritoneal macrophages in the peritoneal cavity. Groups of mice (*n* = 5) were given a single intraperitoneal injection with LPS (10 µg) or PBS as a control, and 3 h later the abundance of macrophages, neutrophils and monocytes in peritoneal lavages was anaylsed by flow cytometry. cDC, conventional dendritic cells; LPM, large peritoneal macrophages; SPM, small peritoneal macrophages. **(A)** Gating strategy. **(B)** Total numbers of CD102 + LPM, neutrophils, B cells and T cells. **(C)** Total numbers of Ly6Chi monocytes, CD11c + SPM, cDC1, cDC1 and eosinophils. Data points represent individual mice. Bars, median. *, *P* < 0.05; **, *P* < 0.01, Student’s t-test.

To test this hypothesis, groups of mice were treated with LPS (10 µg) or PBS as a control, and 3 h later the abundance of macrophages and other leukocyte and lymphocyte populations in peritoneal lavages was compared by flow cytometry (Fig. 6A). Our data show that within 3 h of LPS-treatment the retrievable abundance of large peritoneal macrophages was significantly reduced compared to PBS-treated controls (Fig. 6B). A significant reduction in number of peritoneal MHCII^+^ antigen presenting cells was also observed, including CD11b^+^CD11c^−^ small peritoneal macrophages, CD11b^+^ CD11c^+^ cells comprising both CD11c^+^small peritoneal macrophages and cDC2 conventional dendritic cells (cDC)^41,42^ and CD11b^−^CD11c^+^ cDC1 (Fig. 6C). These findings mirror the rapid loss of peritoneal macrophages and DC that has previously been reported following injection of similar sterile pathogen-derived stimuli^43^. In contrast, a small but significant increase in the abundance of neutrophils was observed in the peritoneal cavity after LPS-treatment, whereas monocytes, B cells, T cells and eosinophils were unchanged (Fig. 6B&C).

## Discussion

Here we show that a single IP injection with LPS significantly enhances prion disease susceptibility by approximately 100X when given between 24 and 3 h before peripheral infection by the IP route. The impact of the LPS-exposure on prion disease susceptibility was acute and restricted to the time immediately before infection, since treatment as soon as 3 h after IP prion injection did not affect disease susceptibility. Macrophages are considered to provide protection against infection by engulfing and sequestering the prions. Consistent with this, the retrievable abundance of macrophages within the peritoneal cavity was reduced within 3 h after LPS treatment, and this coincided with the increased early abundance of prions in the spleens of LPS-treated mice at 35 days after exposure. Together, our data suggest that the effects of acute pro-inflammatory stimulation on the reduced retrievable abundance of macrophages in the peritoneal cavity significantly increased disease susceptibility by limiting the ability of macrophages to sequester prions, enhancing the early propagation of the prions from the site of exposure to the spleen.

After peripheral infection, the initial accumulation and replication of prions upon FDC in lymphoreticular tissues such as the spleen is essential for their subsequent spread to the CNS^44^. The temporary ablation of FDC in the spleen up to 42 days after IP infection with prions reduces disease susceptibility and delays survival times^11,40,45^. However, in the current study LPS-treatment as soon as 3 h after prion infection did not affect disease pathogenesis. This suggests that the effects of LPS treatment on disease susceptibility were not due to effects on FDC abundance, size or PrP^C^expression since alterations to each of these would be expected to have impact in the days following an infection. Indeed, the abundance of FDC in the spleen at 35 days after LPS-treatment was similar to controls. This contrasts the reported effects of repetitive immunization with an independent antigen (CpG oligonucleotides followed by BSA/alum) on prion disease susceptibility^46^. Although the direct mechanism was not identified in that study, repeated immunization enhanced susceptibility to IP prion infection, and coincided with the increased density and size of the FDC networks in the spleen at the time of infection.

The propagation of ME7 prions from the site of exposure in the peritoneal cavity to the FDC in the spleen occurs independently of PrP^C^expression in lymphocytes, mononuclear phagocytes and other haematopoietically-derived cells^5,15^. Furthermore, transgenic over-expression of PrP^C^in B cells^47^or T cells^48^ is insufficient to enable prion replication within them. This suggests that the effects of effects of LPS treatment on disease susceptibility are not due to potential effects on PrP^C^ expression in these cell populations.

Acute pro-inflammatory stimulation or bacterial infection in the peritoneal cavity rapidly trigger the macrophage disappearance reaction^21,22^. During this process the peritoneal macrophages are contained within temporary cellular aggregates that attach to the mesothelium, and these are considered to help prevent the dissemination of bacterial infections^23^. As a consequence, macrophages are temporarily no longer retrievable from the peritoneal cavity^23^. Thioglycolate-induced peritonitis may also induce macrophage cell death via apoptosis^23^. In the current study we show that LPS treatment similarly reduced the recovery of macrophages from the peritoneal cavity, and this coincided with the increased early accumulation of prions in the spleen. Macrophages are considered to sequester or destroy prions via lysosomal and proteosomal pathways^24–28^. We therefore consider it is plausible that the reduced availability of macrophages in the peritoneal cavity of LPS treated mice at the time of exposure limited the ability of these cells to sequester prions at the site of exposure. As a consequence, a greater abundance of prions from the original inoculum was able to initially infect the spleen. Data in an independent study have suggested that LPS treatment may transiently impede the ability of in vitro cultivated macrophages to clear prions^49^. Further studies are necessary to determine whether the ability of any remaining macrophages to clear prions in vivo was also impeded in the current study after LPS treatment.

In vitro studies have suggested that LPS can interact with recombinant PrP^C^and induce conversion into an isomer with increase beta-sheet content^50^. Whether LPS can similarly induce the conversion of cellular PrP^C^ into infectious PrP^Sc^ in vivo was not determined in the above study, but if this was a major contribution to the increased disease susceptibility in the current study, we would expect to observe an effect on disease susceptibility when given after prion infection. Analysis of the prion disease specific vacuolation within the brains of the clinically-affect mice was also consistent with infection with ME7 scrapie prions, and did not suggest the presence of a novel, LPS-induced, prion species.

Together, our data suggest that the reduced retrievable abundance of macrophages in the peritoneal cavity following acute LPS-treatment increased disease susceptibility by limiting their ability to sequester prions, which enhanced the efficiency of the initial propagation of the prions from site of exposure (peritoneal cavity) to the spleen from where neuroinvasion subsequently occurs. This conclusion is also consistent with the independent demonstration that macrophages and cDC were the predominant populations that captured fluorescently-labelled prions in the peritoneal cavity^51^. An earlier study has reported that chlodronate-mediated macrophage depletion similarly increased the early accumulation of prions in the spleen after IP exposure^52^. Although effects on disease susceptibility were not studied, the effects of chlodronate-treatment were not restricted to macrophages as it also led to a transient ablation of FDC and B cells in the spleen. Although a small increase in neutrophils was also observed in the peritoneal cavity after LPS treatment in the current study, relatively few of these cells are considered to acquire prions^51^. The effects of repetitive immunization on IP prion disease susceptibility reported by Bremer and colleagues^46^ also coincided with reduced numbers of splenic marginal zone macrophages which may have had the potential to increase the efficiency of the initial delivery of the prions to FDC.

The macrophage disappearance reaction is a transient process. Within days of exposure to the initial stimulus, the mesothelium-attached cellular aggregates resolve and monocytes act to replace the macrophages in the peritoneal cavity^23,39,53^alongside proliferation of the remaining resident cells^54^. Treatment with LPS did not impact on prion disease susceptibility when administered 48 h or more before prion infection. This is consistent with the transient nature of the macrophage disappearance reaction, and the subsequent recovery of the macrophages in the peritoneal cavity.

Independent studies and our previous data have shown that IP LPS administration can acutely affect the status of microglia and astrocytes directly within the CNS^32,55,56^. If the effects of LPS treatment were mediated via transient effects on cells in the brain or the permeability of the blood-brain-barrier, we would expect treatment soon after prion exposure to also affect disease susceptibility. However, our data suggest that direct effects on the CNS are not contributing factors. In the current study we show that IP LPS treatment as soon as 3 h after IP prion infection had no effect on disease susceptibility compared to controls. In a separate study we have also shown that IP LPS treatment, including up to 4 daily doses, soon after the injection of prions directly into the brain by intracerebral injection similarly has no influence on the subsequent development of CNS prion disease^32^. Together, data in our previous study^32^ and presented here suggest that any potential acute effects of LPS treatment on the brain around the time of prion exposure, are not responsible for the increased disease susceptibility described in the current study. This implies that the effects of LPS on disease susceptibility were due to effects on cells directly in the peritoneal cavity rather than in the CNS.

Together, our data suggest that a reduced abundance of macrophages at the site of infection enhances susceptibility to a peripherally-acquired prion infection. Our findings are consistent with the suggestion that macrophages play a host-protective role during infection by engulfing and sequestering prions. These data also help to advance our understanding of how factors that reduce the abundance of macrophages around the time of peripheral exposure to prions may increase disease susceptibility by enhancing the propagation of the prions from sites of peripheral exposure to the lymphoid tissues from where neuroinvasion subsequently occurs. Distinct populations of mononuclear phagocytes are also present within the CNS^57^. Macrophage containing aggregates may also form upon the epithelial surfaces of other body cavities such as the brain ventricles in response to acute inflammation or bacterial infection, leading to a similar transient reduction in macrophage abundance at these sites^23^. Whether a reduction in the abundance of intraventricular macrophages might increase the burden of prions in the brain is uncertain. Further studies may help to identify novel macrophage-targeted treatments that can enhance this process and reduce susceptibility to peripherally acquired prion infections.

## Data Availability

All data supporting the conclusions of this study are available within the article, or from the corresponding author upon reasonable request.
